# Genetic Architecture of Pediatric Cardiomyopathies Assessed by Whole‐Exome Sequencing: Insights Into Early‐Onset and Syndromic Forms

**DOI:** 10.1111/cge.70230

**Published:** 2026-08-13

**Authors:** Luana Giovannangeli, Elise Daire, Kahia Messaoudi, Didier Herent, Nathalie Desjeux, Emilie Lacot‐Leriche, Sarah Sauval, Sabine Dirani, Pascal De Groote, Didier Klug, Jean‐Benoit Baudelet, Alexandre Delarue, Olivia Domanski, Pierre‐Alexandre Fontanges, Jamal Ghoumid, Luisa Marsili, Alexis Hermida, Florence Jobic, Guillaume Jedraszak

**Affiliations:** ^1^ Laboratoire de Génétique Constitutionnelle CHU Amiens‐Picardie Amiens France; ^2^ Service de Cardiologie et Pneumo allergologie pédiatriques CHU Amiens‐Picardie Amiens France; ^3^ UR4666 HEMATIM, équipe CARDIOMICS, Centre Universitaire de Recherche en Santé Université de Picardie Jules Verne Amiens France; ^4^ Service de Génétique Clinique CHU Amiens‐Picardie Amiens France; ^5^ Service de Cardiologie CHU de Lille Lille France; ^6^ Service de Cardiologie infantile et congénitale CHU de Lille Lille France; ^7^ Clinique de Génétique Guy Fontaine Lille France; ^8^ Université de Lille, ULR7364 RADEME, Hôpital Universitaire de Lille Lille France; ^9^ Department of Genetics University Medical Center Utrecht, Utrecht University Utrecht the Netherlands; ^10^ Service de Cardiologie CHU Amiens‐Picardie Amiens France

**Keywords:** cardiomyopathy, pediatrics, retrospective cohort study, whole exome sequencing

## Abstract

Pediatric cardiomyopathies (CM) are rare and heterogeneous heart disorders, including hypertrophic, dilated, restrictive, arrhythmogenic, and non‐dilated CM. While their genetic basis is well characterized in adults, it remains less clearly defined in children particularly in early‐onset apparently isolated and syndromic forms. We conducted a retrospective study (2018–2024) of 59 pediatric patients who underwent Whole‐Exome Sequencing (WES) for CM at Amiens and Lille University Hospitals, aiming to characterize the genetic architecture of pediatric CM. WES identified at least one Variant Of Interest (VOI) in 62.7% of patients (37/59), including 45.8% (27/59) of P/LP variants, and 16.9% (10/59) of VUS. VOI identification yields for HCM and DCM were respectively 67.7% and 57.1%. Yield was higher in patients diagnosed before 1 year of age compared with those diagnosed later (72.7% vs. 56.7%). Variants were identified in classical CM genes, including sarcomeric genes, but also in less typical and syndromic genes. Among patients diagnosed before 6 months, 55% carried a variant in genes associated with syndromic cardiomyopathy, including cases with initially isolated cardiac phenotypes. These findings support the use of WES as a first‐line approach in pediatric CM and highlight the contribution of complex and syndromic genetic architectures to early‐onset and severe diseases.

## Graphical abstract

Pediatric cardiomyopathies (CM) are a heterogeneous group of disorders. Their genetic basis remains poorly defined, particularly in children with early‐onset and apparently isolated forms. With a diagnostic yield of 62.7%, our findings suggest that whole‐exome sequencing could improve the diagnosis, genetic counseling, and clinical management of pediatric CM.

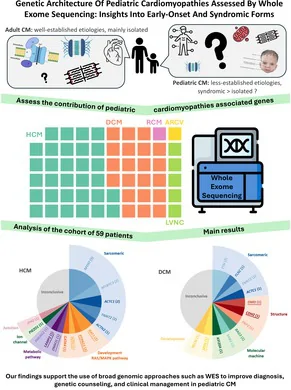

## 1 Introduction

Pediatric cardiomyopathies (CM) are a heterogeneous group of myocardial disorders that include hypertrophic (HCM), dilated (DCM), restrictive (RCM), arrhythmogenic (ARVC), and non‐dilated left ventricular cardiomyopathies. Recent revisions have redefined left ventricular non‐compaction (LVNC) as a phenotypic trait rather than a distinct condition [1]. Although rare, affecting 1.2 per 100 000 children over 10 years and 8 per 100 000 children under 1 year [2], pediatric CM are associated with rapid progression, substantial morbi‐mortality and remain a leading indication for heart transplantation in childhood [3].

The genetic basis of CM has been extensively investigated in adults, where pathogenic variants in sarcomeric genes account for 40%–60% of cases [4]. Consequently, current genetic testing strategies often rely on targeted gene panels [1]. While effective for identifying variants in well‐established cardiomyopathy genes, these approaches may overlook atypical, syndromic, or unexpected genetic etiologies [5].

In children, the genetic architecture of CM appears more complex than in adults. Vasilescu et al. [6] reported that 40 of 66 affected children lacked a molecular diagnosis, and Xing et al. [7] described diagnostic yields ranging from 33.3% to 72.7% depending on CM subtype. Beyond classical sarcomeric forms, pediatric presentations may be associated with metabolic disorders, developmental syndromes, or other multisystem diseases, sometimes preceding the appearance of extracardiac manifestations. Comprehensive genomic approaches such as Whole‐Exome Sequencing (WES) may therefore be particularly valuable in this population by enabling the simultaneous analysis of both established cardiomyopathy genes and a broader spectrum of disease‐associated genes [6, 7].

In this study, we investigated the genetic architecture of pediatric CM in a cohort of 59 patients using WES. We assessed the contribution of CM‐associated genes, determined the prevalence of Pathogenic (P), Likely Pathogenic (LP), and Variants of Uncertain Significance (VUS), with particular attention to the contribution of syndromic etiologies and early‐onset disease.

## 2 Methods

### 2.1 Study Population

We conducted a retrospective study of pediatric patients (< 18 years) diagnosed with CM and referred for genetic testing between January 2018 and September 2024. The study was approved by the institutional ethics committees (n°PI2024_843_0057). Patients were recruited from Amiens and Lille University Hospitals. Inclusion criteria were confirmed CM with onset before 18 years, availability of complete clinical and sequencing data, and informed consent. Patients with uncertain phenotypes were excluded. Information on family history of CM was requested but it was not mandatory to benefit from WES. CM were classified according to the 2015 European Society of Cardiology guidelines.

### 2.2 Genetic Analyses

Solo WES was performed using SureSelect Human All Exon v7 or v8 (Agilent technologies) on a NextSeq550 system (Illumina). Bio‐informatic analyses were performed on the SeqOne platform using the hg19 reference genome. Coding and ±10 bp intronic variants with a minor allele frequency (MAF) < 10^−5^ in GnomAD [8] and < 0.001 in our internal database (2500 samples) were filtered. We prioritized genes associated with cardiac phenotype (Cardiomyopathy, HP:0001638) according to the Human Phenotype Ontology (HPO) [9]; 604 genes were associated with this phenotype (Data S2). Candidate variants were further evaluated using gnomAD, ClinVar, OMIM, and in silico prediction tools (SIFT, REVEL, MPA, CADD, SpliceAI, and SPiP). Variants were classified according to ACMG–AMP guidelines [10], by two independent molecular geneticists. Any discordant classifications were adjudicated by a multidisciplinary team. P/LP/VUS were validated by Sanger sequencing, and segregation and phasing analyses were performed when feasible and relevant.

## 3 Results

### 3.1 Study Population

Among 85 initially unrelated eligible probands, 26 were excluded (uncertain diagnosis, lack of consent, or age > 18 years), leaving 59 probands for analysis (Figure 1). HCM was the most frequent subtype (31/59), followed by DCM (21/59), LVNC (5/59), ARVC (1/59), and RCM (1/59). Five patients had a first‐degree family history of CM (four HCM, one LVNC) and one patient had family history of DCM at the second degree. Median age at diagnosis was 4 years [IQR: 0.5–13]. DCM was diagnosed earlier than HCM (2 years [IQR: 0.3–4] vs. 8 years [IQR: 0.54–14] respectively), likely reflecting prenatal detection in three DCM cases. In HCM, diagnoses were equally distributed before and after 10 years (16/31 vs. 15/31).

**FIGURE 1:**
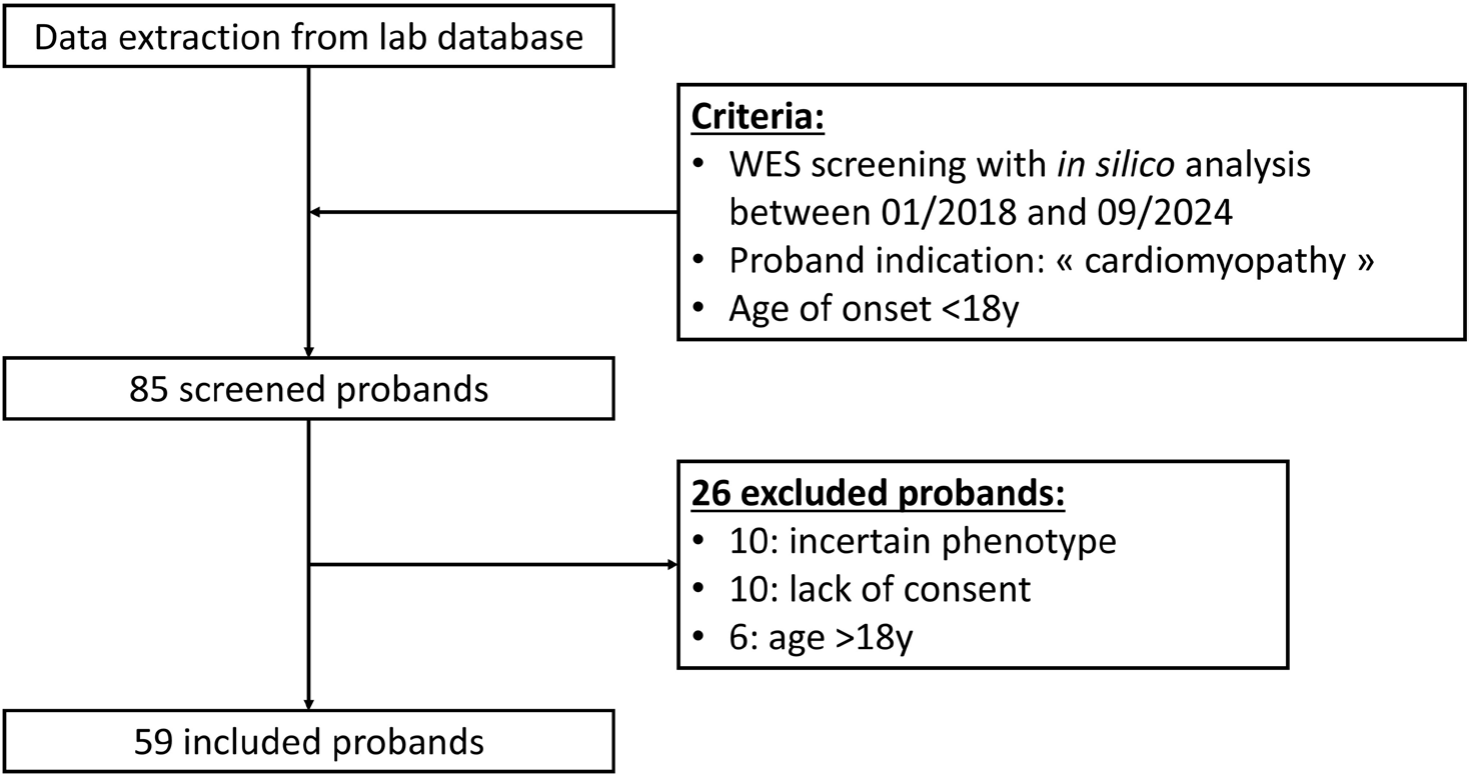
Flowchart.

### 3.2 Genetic Diagnostic Yield

WES identified at least one Variant of Interest (VOI) P, LP, or VUS in 37/59 patients (62.7%), of whom 45.8% (27/59) had P/LP variants and 16.9% (10/59) had VUS (Data S1). Yield was higher in patients diagnosed before 1 year (16/22, 72.7%) than after (21/37, 56.7%). Five patients carried heterozygous variants in two different genes, suggesting a potential combined effect on phenotype severity. In four additional cases, a single P/LP variant was considered insufficient to explain the phenotype, with no second hit identified.

### 3.3 Subtype‐Specific Findings

The yield of VOI was 67.7% for HCM and 57.1% for DCM. P/LP variants were more frequent in HCM, whereas VUS predominated in DCM. Among HCM patients, 21/31 (67.7%) carried a VOI, including 16/31 (51.6%) carried P/LP variants. Most of these variants involved sarcomeric genes, with *MYH7* being the most frequently implicated (*n* = 5) (Figure 2). Variants in metabolic genes (*LAMP2*, *PRKAG2*) and RAS/MAPK pathway genes were also identified. Among DCM patients, 12/21 (57.1%) had VOI, including 7/21 (33.3%) with P/LP variants. Three patients with a DCM showed an added LVNC phenotype. Sarcomeric genes accounted for one‐third of findings (*TTN*, *FLNC*, *TNNT2*, *ACTC1*), and we also identified variants in less typical genes (*SPEG*, *KIF20A*, *MIB1*). One probable incidental CNV (Xp21.2p11.4 deletion including *DMD* gene) was detected (Figure 2). Among other subtypes, two LVNC patients carried dual variants (*MYH7*/*MYH7* in cis; *MYH7*/*LMNA*), the RCM patient carried heterozygous variants in *MYH7* and *CACNA1C*, and the ARVC patient harbored a *PKP2* variant.

**FIGURE 2:**
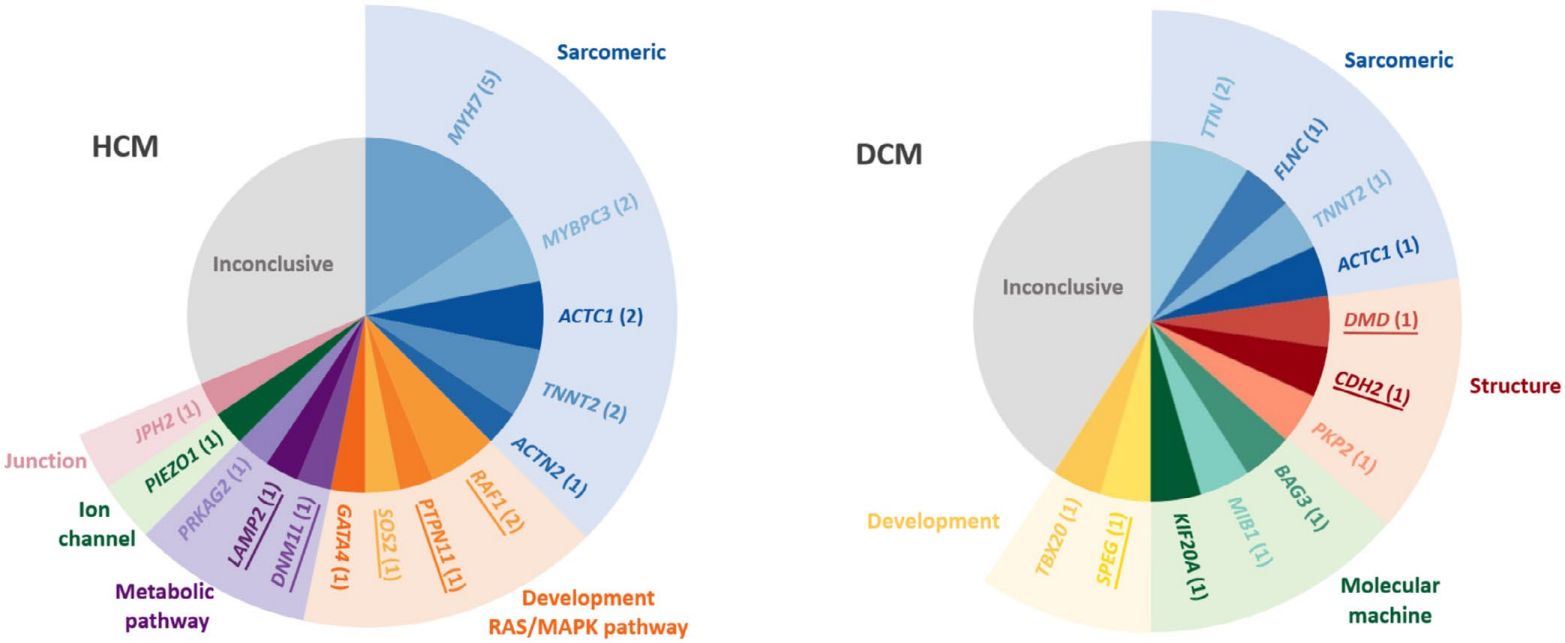
Genes implicated in HCM (left) or DCM (right). Two patients carry co‐occurring variants in more than one gene: MYH7 + PIEZO1 in one patient with HCM, and TTN + TNNT2 in one patient with DCM. Underlined genes are associated with syndromic cardiomyopathies.

### 3.4 Syndromic Cardiomyopathies

Variants in syndromic genes were identified in 9 patients (15%) and were associated with earlier onset of CM (median 0.4 [IQR: 0.17–1.5] vs. 4 years [IQR: 0.86–15], *t*‐test *p* = 0.056). Among all patients diagnosed with a CM before 6 months, 55% (6/11) carried a variant in a syndromic gene. In HCM and DCM (*n* = 52), syndromic variants accounted for 17.3% (9/52) and were absent in other subtypes (Figure 3). Notably, half of these patients initially presented with isolated or nonspecific phenotypes. Four HCM patients carried RAS/MAPK variants typically associated with Noonan syndrome despite subtle initial features, and one patient with a *SPEG* variant presented with apparently isolated CM at 2 months.

**FIGURE 3:**
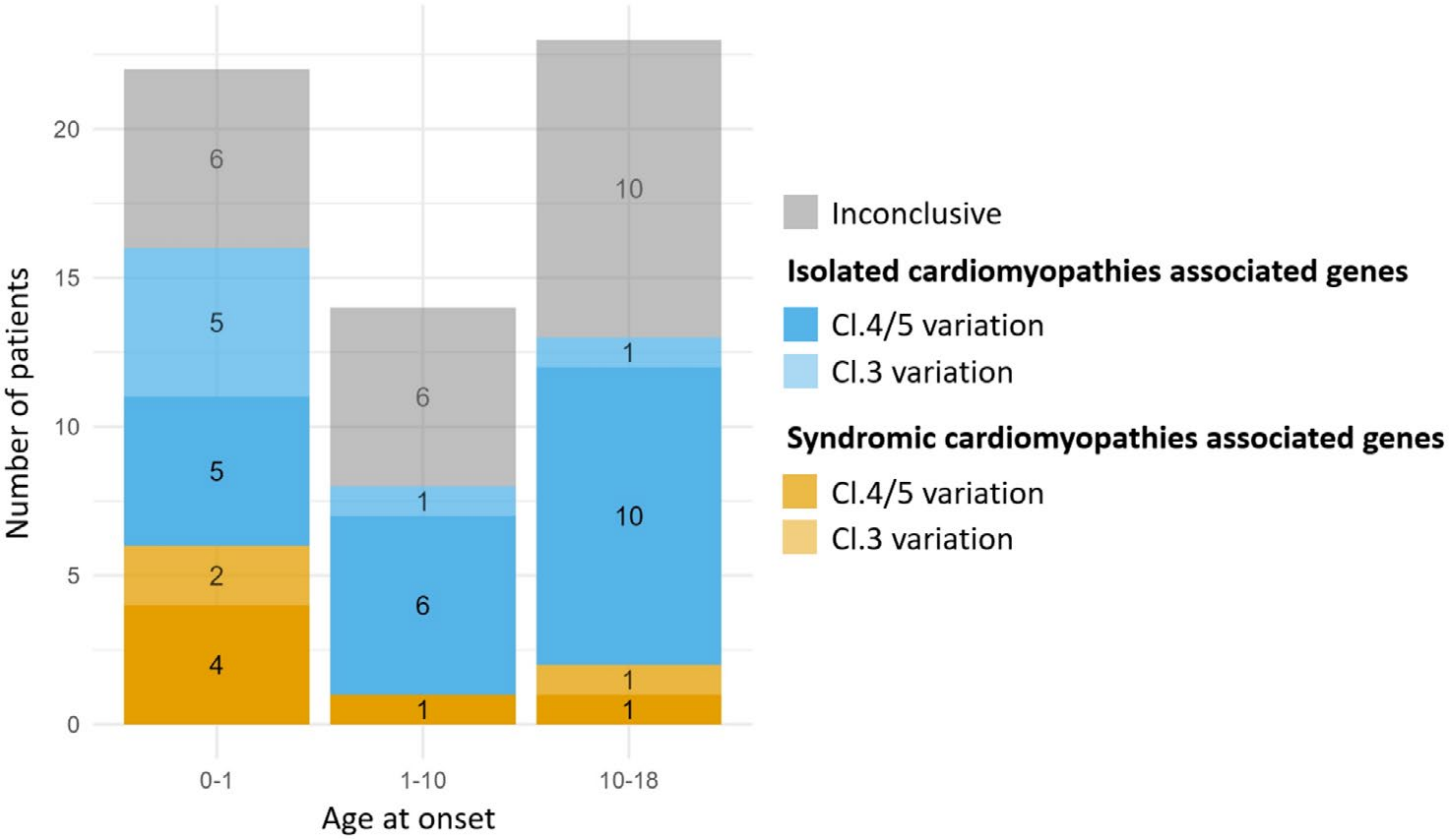
Number of patients with inconclusive WES analysis or carrying at least one Class 3, 4, or 5 variants in a gene associated with syndromic (orange) or isolated (blue) cardiomyopathy, by age group (0–1, 1–10, and 10–18 years).

## 4 Discussion

Our study included 59 pediatric patients referred for molecular diagnosis after a diagnosis of pediatric cardiomyopathy. Performing WES, we identified at least one VOI in 62.7% of patients.

This diagnostic rate is higher than those reported in previous pediatric WES cohorts. Ware et al. [11] reported a rate of 56% in 528 patients, and Keisling et al. [12] reported a rate of 39.6% in 91 patients. Diagnostic yields in panel‐based studies ranged from approximately 40% with targeted panels [6, 13] to 53.7%–60.4% with broader testing strategies incorporating multiple variant types, including SNVs, CNVs, and chromosomal abnormalities [14, 15]. Overall, WES‐based approaches tend to achieve higher diagnostic yields than panel‐based strategies due to broader genomic coverage and detection of atypical or unexpected variants.

When examined by subtype, diagnostic yields were comparable between HCM and DCM (67.7% and 57.1%, respectively), consistent with previous pediatric studies and in contrast to adult cohorts where HCM has a highly better yield [14, 15]. Sarcomeric genes accounted for most variants in HCM, with *MYH7* (23.8%, 5/21) and *MYBPC3* (9.5%, 2/21) being the most frequently implicated genes. In contrast to adult series where *MYBPC3* predominates, *MYH7* was the most frequently implicated gene in our series. In the literature, *MYH7* and *MYBPC3* variants together account for 40% of isolated HCM and up to 70% of familial forms [16]. The genetic architecture of DCM was more heterogeneous, involving both classical cardiomyopathy genes (*TTN*, *FLNC*, *TNNT2*, *ACTC1*) and less typical disease‐associated genes such as *SPEG*, *KIF20A*, and *MIB1*, supporting a greater contribution of rare or atypical loci in pediatric DCM compared with HCM [11, 15].

Beyond subtype‐specific patterns, syndromic etiologies represented a key component of the genetic landscape in our cohort with 9 patients carrying a variation in a gene associated with syndromic CM. Variants involved metabolic disorders or signaling pathways (*LAMP2, DNM1L, PRKAG2*) and RAS/MAPK signaling genes, with Noonan‐spectrum disorders identified in 12.9% (*n* = 4/31) of HCM cases. Molecular diagnosis is particularly important in these patients given the severity of Noonan‐associated HCM, especially during infancy [17], and the emergence of targeted therapies such as MEK inhibition [18]. These findings illustrate the etiological diversity of pediatric cardiomyopathy and the greater contribution of syndromic causes compared with adult disease [6, 13]. Notably, 6 patients carrying syndromic variants (*n* = 9) initially presented with apparently isolated cardiomyopathy, highlighting the limitations of phenotype‐based prediction.

Age at onset strongly influenced diagnostic yield. Patients diagnosed before 1 year had a higher yield (16/22, 72.7%) than those diagnosed later (21/37, 56.7%). Consistent with this finding, cardiomyopathies associated with syndromic genes were diagnosed earlier (median 0.4 years [IQR: 0.17–1.5]) than isolated forms (4 years [IQR: 0.86–15]).

Although early‐onset cardiomyopathy is classically associated with syndromic diseases, 9 patients in our cohort presented with early or severe disease despite carrying variants typically associated with adult‐onset cardiomyopathies (*MYBPC3*, *MYH7*, *TTN*). Such variability is well documented, particularly in adult studies, with marked intra‐familial differences in age at onset and severity [19, 20]. Several mechanisms such as de novo variants and oligogenic inheritance are known in the literature and may contribute to such presentations [6, 21, 22]. In our cohort, five of the nine early/severe patients carrying sarcomeric variants also harbored a second VOI, of whom two carried two P/LP variants. Moreover, a single variant in genes traditionally associated with adult‐onset disease may be insufficient to explain early or severe phenotypes, further highlighting the genetic complexity of pediatric cardiomyopathy.

Our study has several limitations. Phenotypic analyses relied on clinical information available at the time of testing and may have missed evolving features. While WES enables broad genetic exploration it does not capture all genomic regions, does not sequence mitochondrial DNA, and may include areas of low coverage, thereby limiting variant detection. Moreover, solo WES limits the assessment of inheritance patterns. The retrospective design and relatively small sample size (*n* = 59) reduce the generalizability of our findings and the power of subtype‐specific analyses. Finally, incomplete familial investigations limited the interpretation of segregation and penetrance.

In conclusion, our study highlights the benefit of WES in pediatric cardiomyopathy, with a diagnostic yield of 62.7% and improved detection of rare, syndromic and complex genetic etiologies. Early‐onset CM, particularly before 1 year of age, emerged as a key indicator of genetic etiology, enriched in syndromic forms. These findings support the use of broad genomic approaches to improve diagnosis and clinical management in CM.

## Funding

The authors have nothing to report.

## Conflicts of Interest

The authors declare no conflicts of interest.

## Supporting information


**Data S1:** Clinical characteristics, variants of interest, and validation criteria for 37 patients of the cohort.

**Data S2:** List of genes associated with Cardiomyopathy (HP:0001638) according to the Human Phenotype Ontology (HPO).

## Data Availability

The data that supports the findings of this study are available in the Supporting Information of this article.
